# Development and Validation of an Artificial Intelligence Electrocardiogram Recommendation System in the Emergency Department

**DOI:** 10.3390/jpm12050700

**Published:** 2022-04-27

**Authors:** Dung-Jang Tsai, Shih-Hung Tsai, Hui-Hsun Chiang, Chia-Cheng Lee, Sy-Jou Chen

**Affiliations:** 1 Institute of Life Sciences, School of Public Health, National Defense Medical Center, Taipei 11499, Taiwan; oo800217@gmail.com; 2 Department of Emergency Medicine, Tri-Service General Hospital, National Defense Medical Center, Taipei 11499, Taiwan; doc50024@ndmctsgh.edu.tw; 3 School of Nursing, National Defense Medical Center, Taipei 11499, Taiwan; sheisvivian@gmail.com; 4 Planning and Management Office, Tri-Service General Hospital, National Defense Medical Center, Taipei 11490, Taiwan; clee112@ndmctsgh.edu.tw

**Keywords:** electrocardiogram, triage, emergency department, artificial intelligence, machine learning, decision support system

## Abstract

The machine learning-assisted electrocardiogram (ECG) is increasingly recognized for its unprecedented capabilities in diagnosing and predicting cardiovascular diseases. Identifying the need for ECG examination early in emergency department (ED) triage is key to timely artificial intelligence-assisted analysis. We used machine learning to develop and validate a clinical decision support tool to predict ED triage patients’ need for ECG. Data from 301,658 ED visits from August 2017 to November 2020 in a tertiary hospital were divided into a development cohort, validation cohort, and two test cohorts that included admissions before and during the COVID-19 pandemic. Models were developed using logistic regression, decision tree, random forest, and XGBoost methods. Their areas under the receiver operating characteristic curves (AUCs), positive predictive values (PPVs), and negative predictive values (NPVs) were compared and validated. In the validation cohort, the AUCs were 0.887 for the XGBoost model, 0.885 for the logistic regression model, 0.878 for the random forest model, and 0.845 for the decision tree model. The XGBoost model was selected for subsequent application. In test cohort 1, the AUC was 0.891, with sensitivity of 0.812, specificity of 0.814, PPV of 0.708 and NPV of 0.886. In test cohort 2, the AUC was 0.885, with sensitivity of 0.816, specificity of 0.812, PPV of 0.659, and NPV of 0.908. In the cumulative incidence analysis, patients not receiving an ECG yet positively predicted by the model had significantly higher probability of receiving the examination within 48 h compared with those negatively predicted by the model. A machine learning model based on triage datasets was developed to predict ECG acquisition with high accuracy. The ECG recommendation can effectively predict whether patients presenting at ED triage will require an ECG, prompting subsequent analysis and decision-making in the ED.

## 1. Introduction

An electrocardiogram (ECG) is a noninvasive and readily available tool that provides vital information about cardiovascular diseases such as acute coronary syndrome, arrhythmia, and hemodynamic instability in the emergency department (ED). Guidelines for acute coronary syndrome (ACS) suggest an ECG be taken within 10 min as an initial step of risk stratification to identify high-risk patients for timely management [1,2]. Therefore, most ED triages have pre-established screening criteria, such as acute chest, epigastric pain and pressure, or pain radiating to the jaw or left arm, to identify patients who should receive an immediate ECG examination. Other than that, the decision for an ECG examination is generally symptoms-based and driven by physicians. Thus, the time to complete an ECG acquisition in the ED depends on when a patient’s assessment by a physician started, except for patients with cardiac chest pain, who are prioritized for immediate ECG examination once certain pre-established rules of ACS criteria are met at triage [3,4,5,6].

Currently, there is no clinical decision tool for ECG acquisition in conditions other than ACS. For example, the rule-based rapid ECG by Graff et al. [7]; the history, ECG, age, risk factors, and troponin (HEART) pathway [8]; the prioritization rule for an immediate ECG by Glickman et al. [4]; and the Emergency Department Assessment of Chest Pain Score (EDACS) [9] are designed specifically for ACS and are not intended to cover all the other indications. Several ED triage systems, such as the emergency severity index [10], Manchester Triage System (MTS) [11], and Canadian Triage and Acuity Scale (CTAS) [12], are designed to assess all complaints and are neither sensitive nor specific as ECG indicators, or even as indicators of ACS [13,14]. Furthermore, in most circumstances, an ECG will only be analyzed when a physician is available, with a delay of likely dozens of minutes to an hour after ED registration, depending on workloads. Effectively coping with this time window may expedite clinical decision-making on identifying insidious abnormalities that are critical during initial assessment or herald a deteriorating patient yet are unrecognized during the ED stay, particularly in an overcrowded ED.

With artificial intelligence (AI) incorporated into digital ECG analysis, the featurization techniques of ECG analysis have substantially outperformed conventional ECG interpretation [15]. Rapid advances in AI-based deep learning algorithms with high diagnostic performance have opened a new frontier for ECG in cardiology and even crossed over into other medical fields. For example, an AI-based 12-lead ECG can identify left ventricular dysfunction [16], mitral regurgitation, aortic stenosis [17,18], and hypertrophic cardiomyopathy with high accuracy [19]. Previously, we demonstrated the superiority of AI-ECG in detecting acute myocardial infarction in the ED [20]. Using deep learning techniques, a 12-lead ECG can assist early identification of certain metabolic disorders, such as hypokalemia in thyrotoxic periodic paralysis, hyperkalemia, and digoxin intoxication [21,22]. Moreover, AI-ECG has been studied as a means of predicting certain risks, with promising results such as predicting ventricular dysfunction in asymptomatic individuals [16], estimating atrial fibrillation risk for a patient with ECG in sinus status [16], predicting HbA1C levels that correlate with the progression of diabetes mellitus [23], and sending alerts for patients at high risk of in-hospital cardiac arrest. Furthermore, by integrating AI-ECG with AI-chest X-ray, a 12-lead ECG could also enhance the stratification of patients with chest pain who are at risk of aortic dissection [24]. These applications of AI-assisted ECG analysis have not only transformed our knowledge of its capabilities, but also underscore its vital value in clinical applications, particularly in emergency settings. To effectively initiate and coordinate AI in a timely manner, an AI-assisted decision support tool for ECG acquisition used upon a patient’s arrival at the ED is indicated to help build a smart ECG surveillance system.

ECG is well-known for its benefits in early identification of patients with acute myocardial infarction. Recent studies also demonstrated that early execution of ECG increases early identification of life-threatening conditions, such as hyperkalemia [25], digoxin intoxication [22], and pneumothorax [26], which prompt immediate medical interventions. The objective of the present study was to develop and validate a tool with which to predict the need for ECG acquisition as the patient arrives at the ED. Using machine learning to analyze relevant ED triage data with respect to ECG acquisition, we developed a model regarding the prediction of ECG acquisition for patients at ED triage. By integrating such a decision support tool, the ED can speed up the time to first ECG acquisition, thus facilitating clinical decision-making and, in combination with active analysis by AI, alerting physicians to potential risks necessitating early intervention.

## 2. Methods

### 2.1. Population

We retrospectively collected data from August 2017 to November 2020 in the ED of a tertiary hospital in Taipei, which has an estimated annual ED volume of 90,000 per year. The study included all adult ED visits with a clear, recorded disposition of either admission or discharge. Patients of age less than 20 years or with incomplete records were excluded. The institutional review board of Tri-Service General Hospital, Taipei, Taiwan approved this study.

**Figure 1:**
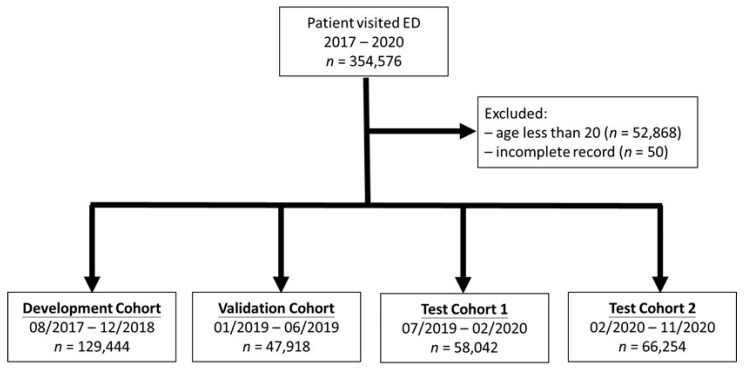
Flowchart diagram of the study cohort generation.

From August 2017 to November 2020, a total of 354,576 patient visits in the ED triage registry were recorded. Among all enrolments, there were 52,868 visits by patients aged less than 20 and 50 visits with incomplete records that were excluded, leaving the analytic cohort of 301,658 ED visits. Data on ED visits were divided into four sets (cohorts), including the development cohort (August 2017–December 2018), the validation cohort (January 2019–June 2019), test cohort 1 (July 2019–6 February 2020), and test cohort 2 (7 February 2020–November 2020, during the COVID-19 pandemic). The test sets were data collected before and during the COVID-19 pandemic, with the onset date defined as 7 February 2020, by Taiwan’s central epidemic command center. A diagram of the sampling process designed to assure a robust and reliable data set of training, validation, and testing for the model development and validation is shown in Figure 1. Once a patient’s data were placed in one of the data sets, those data were not used in other sets.

### 2.2. Data Source

The study included variables of demographics, triage assessment, and chief complaints. Demographic information was either collected at triage or available from electronic health records at the time of the patient encounter, and included age, sex, height, and weight. Triage assessment variables included those routinely collected at triage, such as arrival time, vital signs, and triage acuity levels assigned by the triage nurses. Vital signs included systolic and diastolic blood pressure, pulse, respiratory rate, and body temperature. Thirteen variables of demographics and triage assessment were collected. Chief complaints were recorded in free text format and contained patients’ main descriptions of illnesses collected at triage, which were categorized into 404 different variables using word segmentation technology. We defined the outcome variable as ECG acquisition within 2 h of the ED visit. The detailed information is described in the next section.

### 2.3. Model Training and Development

All data elements were obtained from the enterprise data warehouse, using SQL queries to extract relevant raw data in comma-separated-value format. All subsequent processing was performed in R version 3.4 (R Foundation for Statistical Computing, Vienna, Austria). Below, we summarize the processing steps for each category.

### 2.4. Response Variable

The primary response variable was whether the patient received an ECG exam or not, encoded in a binary variable (1 = done, 0 = none).

### 2.5. Demographic Variable

Thirteen variables in total were included. Values beyond physiologic limits were replaced with missing values. Missing data were imputed using multiple imputations in multivariate analysis [27].

### 2.6. Chief Complaint Variable

Given the high number of unique values (>500) for chief complaints, we classified the main descriptions into 404 variables using jiebaR and encoded them with one-hot encoding.

### 2.7. Model Selection

We trained logistic regression, decision tree, random forest, and gradient boosting (XGBoost) using the development cohort, and decided on a final model based on their accuracy in the validation cohort. All models were trained only once, using default hyperparameters. The R functions “ctree” in the “party” package, “randomForest” in the “randomForest” package, and “xgb.train” in the “xgboost” package were used to implement the above models. The test cohorts were evaluated for the likelihood of ECG acquisition only once by the final model.

### 2.8. Variables of Importance

Information gain is a metric that quantifies the improvement in accuracy of a tree-based algorithm from a split, based on a given variable [28]. We calculated the mean information gain for each variable based on 100 training iterations of the full XGBoost model and listed the top 20 important variables.

### 2.9. Statistical Analysis

The area under receiver operating characteristic curve (AUC) was used to assess model performance, with 95% confidence intervals constructed using the DeLong method implemented in the pROC package [29]. Youden’s index was used to find the optimal cutoff point based on the validation cohort, and we applied it to calculate the sensitivity, specificity, positive predictive value, and negative predictive value for each model [30]. The Kaplan–Meier (KM) method was used to calculate the probability of an ECG acquisition event in the 48 h following admission. The log-rank test was used to test significance.

## 3. Results

### 3.1. Demographics of the Development, Validation, and Test Cohorts

In Table 1, the development cohort comprised 129,444 patient visits, with male sex accounting for 49.8%, a mean age of 53.80, and a mean body mass index of 23.91. Patients in this cohort were predominantly categorized as triage level III (73.5%), followed by triage level II (17.2%). The triage level proportions were relatively comparable among the four cohorts. Trauma visits accounted for less than one-fifth of admissions. The mean pulse rate was 86.9 per minute, the mean respiratory rate was 18.7 per minute, and the mean systolic blood pressure was 135.2 mmHg. In the development set, 33,097 patients had an ECG acquisition within 2 h of their ED admission, accounting for one-fourth of all patient visits, whereas approximately one-third of the patients in the validation and test sets had the examination within 2 h.

### 3.2. Model Development and Validation

**Figure 2:**
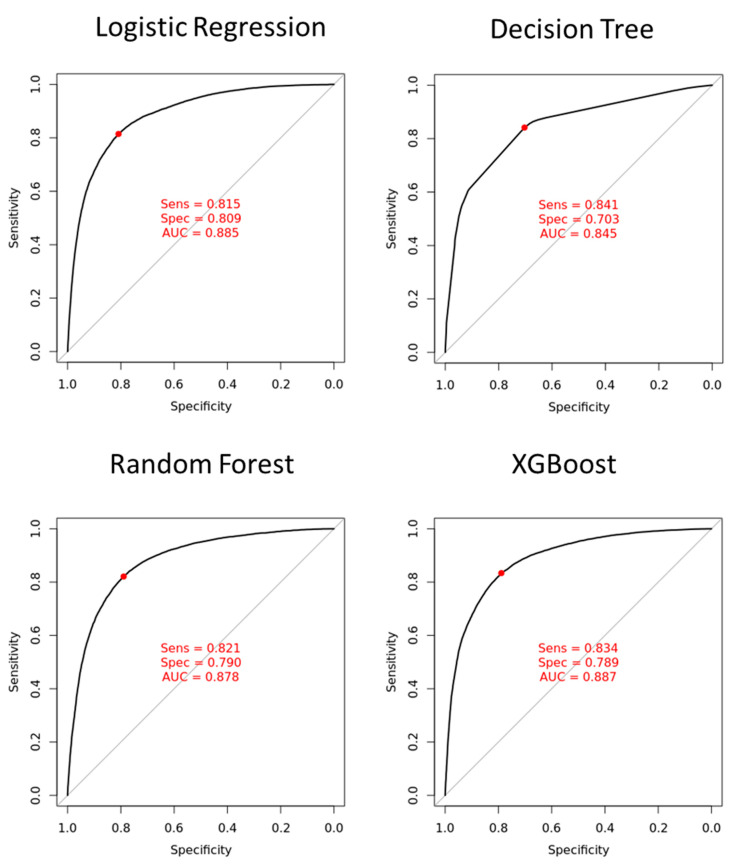
The ROC curves from four prediction models in the validation cohort. The ROC curves (*x*-axis = specificity and *y*-axis = sensitivity) and AUCs were calculated using the validation set. The operating point (red dot) was selected based on the maximum of Youden’s index in the validation cohort, and was used for calculating the corresponding sensitivity and specificity. Sens, sensitivity; Spec, specificity; AUC, area under receiver operating characteristic curve.

Using machine learning of logistic regression, decision tree, random forest, and XGBoost methods, we developed four models and compared their performance on the validation cohort. As shown in Figure 2, the ROC curve demonstrated that the XGBoost model had the highest discriminatory ability (AUC 0.887), followed by the logistic regression model (AUC 0.885), the random forest model (AUC 0.878), and the decision tree model (AUC 0.845). Therefore, we selected XGBoost as the final model of choice for further validation in the two test cohorts.

### 3.3. Performance of the XGBoost Model in the Test Cohorts

**Figure 3:**
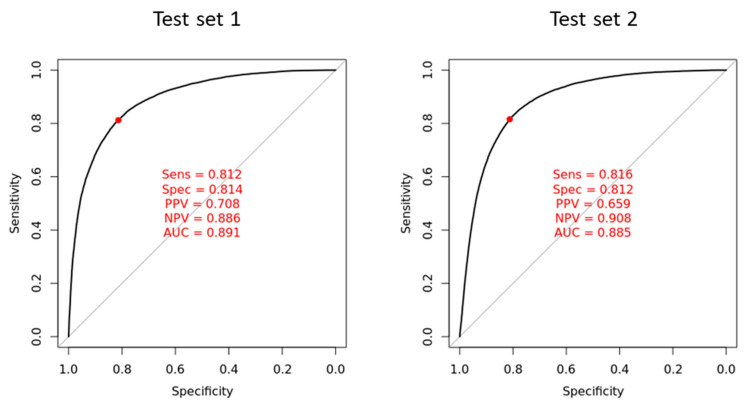
The ROC curves from XGBoost model in two test cohorts. The ROC curves (*x*-axis = specificity and *y*-axis = sensitivity) and AUCs were calculated using the testing sets. The operating point was selected based on the maximum of Youden’s index in the validation cohort, and was used for calculating the corresponding sensitivity and specificity.

As XGBoost outperformed the other three models in 2 h ECG prediction, we further examined its performance in two test cohorts. As shown in Figure 3, the AUC results for predicting 2 h ECG in the XGBoost model were 0.891 in test cohort 1 and 0.885 in test cohort 2, with sensitivities of 0.812 and 0.816, and specificities of 0.814 and 0.812, respectively. The positive predictive values (PPVs) were 0.708 and 0.659, and the negative predictive values (NPV) were 0.886 and 0.908, for test cohorts 1 and 2, respectively, suggesting that the XGBoost method had good discriminatory ability. Relevant parameters of the model are shown in Table 2.

### 3.4. Variable Significance in the XGBoost Model

**Figure 4:**
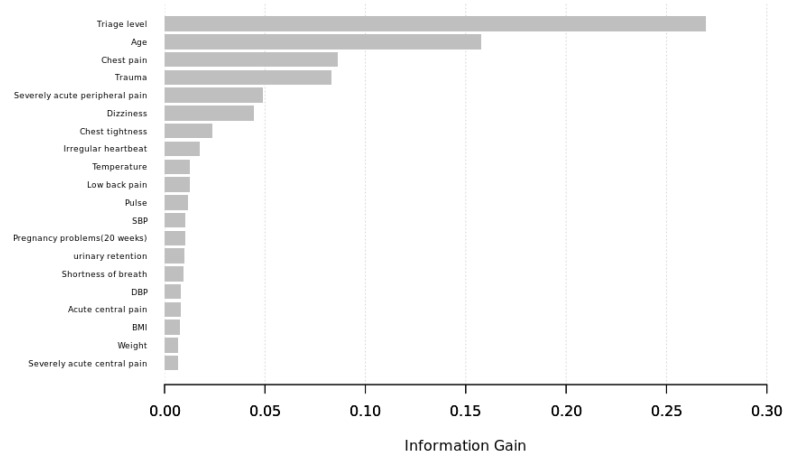
Top 20 variables of significance in the XGBoost model (information gain).

To gain insights into the relevance of each predictor, the top 20 variables of significance for ECG acquisition in the XGBoost model were listed (Figure 4). These were, following the ranking, triage level, age, chest pain, trauma, severely acute peripheral pain, dizziness, chest tightness, irregular heartbeat, temperature, low back pain, pulse, systolic blood pressure (SBP), pregnancy greater than 20 weeks, urinary retention, shortness of breath, diastolic blood pressure (DBP), acute central pain, BMI, weight, and severely acute central pain.

### 3.5. ECG Acquisition in Initially ECG Non-Acquisition Patients Stratified by the AI Model

**Figure 5:**
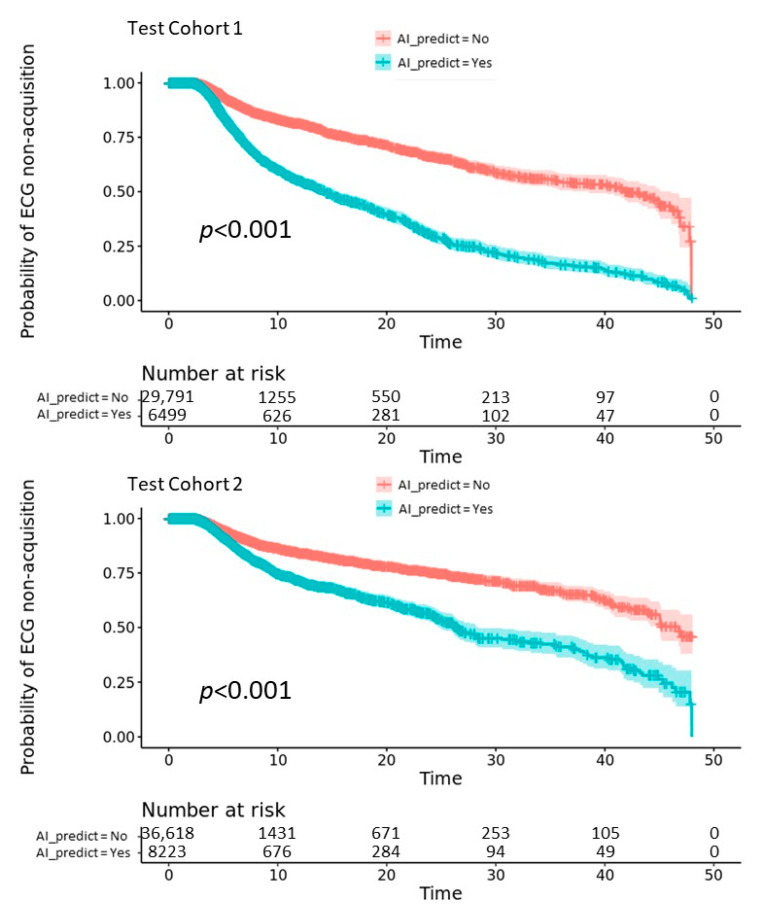
ECG acquisition in initially ECG non-acquisition patients stratified by AI model. The red line demonstrates patients correctly stratified by AI model (true negative), and the blue line demonstrates patients incorrectly stratified by AI model (false positive). The ordinate shows the cumulative probability of non-acquisition of an ECG and the abscissa indicates hours from time of the ED admission.

In Figure 5, we used Kaplan–Meier analysis to compare the difference between positively and negatively AI-predicted groups in those not receiving ECG examinations within 2 h in the two test cohorts. In test cohort 1, out of 36,290 ED visits, 6499 were positively predicted for an ECG acquisition yet did not initially include the examination. The cumulative probability of non-acquisition of an ECG within 48 h of follow-up (false positives) was significantly lower in this group compared with those negatively predicted (true positives) (*p* < 0.001). In test cohort 2, the samples collected during the COVID-19 pandemic, out of 44,841 ED visits, 8233 were positively predicted for an ECG acquisition yet did not receive it, and the cumulative probability of non-acquisition within 48 h of follow-up remained significantly lower in the positively predicted group (20% vs. 50%, *p* < 0.001).

## 4. Discussion 

**Figure 6:**
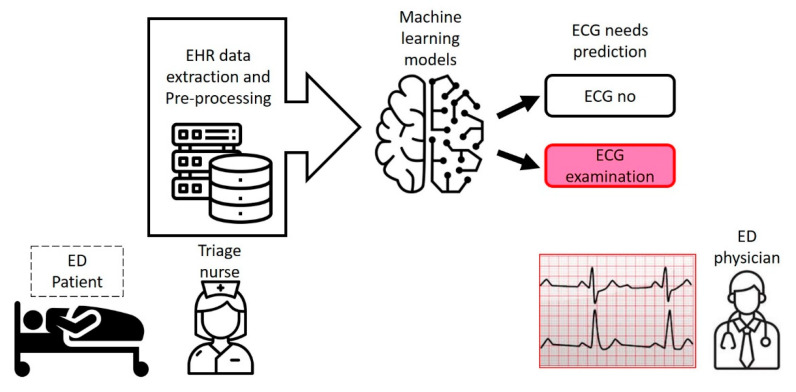
Scheme of machine learning models for ECG decision support at ED triage.

We have developed a triage-based ECG acquisition prediction tool from a cohort of more than 120,000 ED visit samples, reaching an AUC of 0.89, in a single hospital. In two test cohorts, the ECG decision model demonstrated consistent performance, with AUCs of 0.89 and 0.88, respectively, suggesting that an ECG acquisition for ED patients can be predicted with high accuracy at initial encounter using triage data input. Among the variables in the prediction model, triage acuity, age, and chest pain were, in descending order, the three most important predictors of a need for ECG acquisition. Machine learning techniques on ED triage assessment can provide real-time clinical decision support for ECG acquisition with high accuracy (Figure 6).

Machine learning techniques have been widely applied in varied medical fields in prioritizing patients for specific fast healthcare services, such as triage, disease detection, prediction, and classification [31]. To our knowledge, this is the first study to design a decision support tool for predicting the need for ECG acquisition, using machine learning techniques to analyze ED triage data. The prediction tool is designed to help connect triage-based ECG acquisition to an integrated AI-assisted ECG analysis that may aid the decision-making that leads to the early identification of critical conditions. The major advantage of using a triage data system for predictive modeling is that these data are immediately available once a patient arrives at the ED, where clinically relevant information can be obtained for estimating the odds of ECG acquisitions for timely responses. Moreover, in the modeling of validation and testing processes, we examined large data sets of more than 170,000 ED visit samples, testing and comparing four different prediction models and identifying the XGBoost model as having the best discriminatory ability. XGBoost has been considered a gradient boosting method that not only gives great performance and accuracy in both regression and the classification of tabular data, but can also quickly run multiple training cycles while tuning the hyperparameters [32,33]. The decision support tool will respond to information automatically, thus avoiding adding calculations or cumbersome checklist screening to the already-heavy clinical burdens of ED personnel.

An AI-assisted ECG recommendation tool can be a critical element of an ED with an intelligent decision support system (IDSS). With the integration of machine learning and modern computing, the decision support system has evolved to supply smart behavior and support users’ decision-making interactively [34]. The IDSS can learn from previous experiences, recognize the relative importance of certain elements of the decision, gather information, and respond spontaneously according to predefined authorization of the decision-making algorithms, which can potentially improve efficiency and play a critical step in building up a smart ED. Many of the ED patients may present with non-chest pain or atypical symptoms of cardiovascular or pulmonary diseases, such as painless aortic dissection [35], painless acute coronary syndrome, or coexistence of acute myocardial infarction and aortic dissection [36], which are challenging to physicians and warrant earlier identification and clarification for appropriate treatment. By interrogating the need for ECG in real time with the IDSS, the system can signal conditions of relevant risk early. Moreover, AI-aided analysis of ECG can predict heart failure, pulmonary embolisms, electrolyte imbalances, and high risk of mortality. Early response to ECG acquisition can initiate subsequent decisions and diagnosis to identify these potentially critical conditions while managing them with prompt interventions.

Time to an ECG acquisition is indicative of the clinical relevance of time-sensitive characteristics. Hence, we defined the number of patients receiving an ECG within 2 h of arrival as the outcome in developing the model. This minimized the number of ECGs acquired due to conditions other than presenting illnesses or to admission routines. Overall, the model had a precision close to 90%, with sensitivity and specificity over 80%. Moreover, in the follow-up analysis of patients who did not acquire an ECG within 2 h of the initial encounter, those who were positively predicted by the model were significantly more likely to receive an ECG examination within 48 h, further suggesting the model’s robust discriminative ability. Of note, despite the comparable performance of the prediction model on the two test cohorts, the predictive precision on test cohort 2 was slightly inferior to that on test cohort 1. The differences between these two cohorts are likely attributable to temporal differences in the composition of ED patients before and during the COVID-19 pandemic. Contrary to the situation in many other countries, in Taiwan the number of ED visits for febrile conditions dropped by 30 percent during the COVID-19 pandemic [22]. The pandemic also decreased the number of low-acuity ED visits and shortened lengths of stay at the ED. There have also been reports of delayed medical attention for chest pain during the pandemic, leading to poor prognosis in myocardial infarction cases [37]. The public panic and changes in ED patient compositions might have reduced the model’s precision.

The model included vital signs, triage acuity, and chief complaints as predictive variables that are readily available in ED records. The inclusion of comorbidity factors could sensibly improve the model’s fitness; however, data on these variables were frequently incomplete or not available at the initial presentation, and thus, we did not include the features into model training. Among all predictors in the prediction model, the triage acuity variable gained the highest weight, and age ranked as the second most important variable. Patients with increased disease acuity and age often require more medical attention, and an ECG is often an indispensable examination. In addition to cardiopulmonary symptoms and trauma, features of pain-associated conditions were also weighted higher in information gain among others in the prediction model, indicating that an early ECG survey was commonly needed in those with pain-related syndromes in the ED. There are growing numbers of studies on ED triage systems using machine learning techniques to predict various outcomes such as ICU or hospital admission [38,39], length of stay [40], mortality [38], ED revisits [41], and need for critical care [42], with the results mostly suggesting that predictive performance improves with machine learning techniques compared with conventional analysis. By integrating such a decision support tool, the ED may accelerate ECG diagnostic processes by activating an integrated AI-assisted ECG analysis to increase diagnostic proficiency and identify crucial abnormalities early.

Currently, the recommendation tool is not intended to replace physicians’ clinical judgment, but rather to serve as an ancillary tool to facilitate the clinical pathway, particularly in a chaotic ED where task-switching burdens are high during rush hours. The system also does not replace the chest pain protocol at triage, which remains unchanged in its current routines, but rather to work as an adjunct for physicians during patient assessment, recommending those who may need an ECG for subsequent assessment. This strategy would not divert manpower from those in urgent need of an ECG, but serve as a backup reminder to avoid delay in treating those who may otherwise benefit from early ECG interpretation. Therefore, the ECG recommendation can alert ED physicians to obtain ECG results, identify potentially critical diseases or life-threatening conditions, and avoid delayed intervention in the crowded ED.

The main limitation of our study is that the model was derived from a single hospital, limiting its generalizability. Despite the differences in disease distribution among different hospitals, most of the ED triages can be conceptually highly similar, while the variables collected are commonly generalizable. It is reasonable that a hospital should develop its own predictive model based on its own local data to obtain the most precise algorithm. Second, relevant information such as comorbidities and their control status were not captured and included in the assessment. Inclusion of comorbidity data through synchronous acquisition from previous medical records may likely have improved the model’s precision, but at the expense of increased computing load. However, the prediction model was intended to capture characteristics of acute illnesses through a symptom- and acuity-based status immediately collected from registry data for a real-time response. Third, despite being validated with two test cohorts reaching 90 percent accuracy, the model misclassified 10 percent of patient visits. Because the prediction model is heavily weighted by triage acuity, which relies solely on triage nurses’ subjective judgment, and because patients’ initial presenting symptoms are sometimes obscure, assessment variability may exist [34]. Fourth, the model has not been implemented in clinical practice, and its clinical impact, such as on quality of care and outcomes, warrants further study.

In conclusion, an ECG recommendation system can assist clinical decision-making, prompting examinations and activating analyses and timely feedback for physicians. Implementation of an ECG decision support system may enhance decision-making, reducing delay in the chaotic ED environment.

## Figures and Tables

**Table 1:** Characteristics of development, validation, and testing cohorts.

Characteristic	Development	Validation	Testing 1	Testing 2	*p*-Value
Male gender, n (%)	64,462 (49.8)	23,305 (48.6)	28,285 (48.7)	33,857 (51.1)	<0.001
Age (year)	53.80 ± 21.14	53.64 ± 20.91	53.25 ± 20.71	52.09 ± 20.45	<0.001
Height (cm)	163.85 ± 8.90	163.73 ± 8.95	163.81 ± 8.99	164.36 ± 9.00	<0.001
Weight (kg)	64.49 ± 13.92	64.73 ± 14.14	64.80 ± 14.12	65.35 ± 14.23	<0.001
Body mass index (kg/m^2^)	23.91 ± 4.21	24.03 ± 4.26	24.03 ± 4.24	24.07 ± 4.24	<0.001
Temperature (°C)	36.74 ± 0.88	36.69 ± 0.90	36.72 ± 0.90	36.69 ± 0.84	<0.001
Triage level, n (%)					<0.001
I	4672 (3.6)	1786 (3.7)	1974 (3.4)	2073 (3.1)	
II	22,220 (17.2)	8311 (17.3)	9364 (16.1)	9579 (14.5)	
III	95,136 (73.5)	35,222 (73.5)	43,545 (75.0)	45,905 (69.3)	
IV	6385 (4.9)	2242 (4.7)	2739 (4.7)	3072 (4.6)	
V	1031 (0.8)	357 (0.7)	420 (0.7)	5625 (8.5)	
Trauma, n (%)	24,605 (19.0)	8505 (17.7)	10,640 (18.3)	12,308 (18.6)	<0.001
Pulse (beats/min)	86.93 ± 18.86	86.89 ± 18.73	87.43 ± 18.52	86.60 ± 18.56	<0.001
Breath (breaths/min)	18.74 ± 2.68	18.83 ± 2.27	18.72 ± 2.40	18.59 ± 2.00	<0.001
SBP (mmHg)	135.22 ± 24.94	135.96 ± 25.02	134.36 ± 24.55	134.72 ± 24.45	<0.001
DBP (mmHg)	77.86 ± 16.15	76.59 ± 16.08	77.96 ± 15.78	78.55 ± 15.81	<0.001
ECG in 2 h, n (%)	33,097 (25.6)	16,825 (35.1)	20,764 (35.8)	20,380 (30.8)	<0.001

Data were expressed as mean and standard deviation or as numbers with percentage according to data characteristics. SBP, systolic blood pressure; DBP, diastolic blood pressure.

**Table 2:** Model parameters from the validation and test cohorts.

Cohort	Accuracy	Recall	Precision	F Scores
Validation	0.805	0.834	0.681	0.750
Test 1	0.813	0.812	0.708	0.757
Test 2	0.814	0.816	0.659	0.729

## Data Availability

The data presented in this study are available on request from the corresponding author.
